# “I can manage the challenge” – a qualitative study describing experiences of living with balance limitations after first-ever stroke

**DOI:** 10.1080/17482631.2020.1857044

**Published:** 2020-12-16

**Authors:** Mialinn Arvidsson Lindvall, Anette Forsberg, Peter Appelros, Agneta Anderzén-Carlsson

**Affiliations:** a Department of University Health Care Research Center, Faculty of Medicine and Health, Örebro University, Örebro, Sweden; b Department of Physiotherapy, Faculty of Medicine and Health, Örebro University, Örebro, Sweden

**Keywords:** Balance experience, stroke, postural stability, qualitative study, recovery

## Abstract

**Purpose**: To describe experiences of living with balance limitations after first-ever stroke.

**Materials and methods**: This study has a qualitative design, comprising interviews with 19 persons with first-ever stroke, ten women and nine men. Their mean age was 77 years and the mean time since stroke was 15 months. Stroke survivors who were able to walk outdoors with or without a walking aid and who were independent in toileting and dressing were invited to participate. Semi-structured individual interviews were performed. An inductive qualitative content analysis of the manifest and latent content was conducted.

**Results**: The results are presented in two themes illustrating the latent content of the data, “*Feeling dizzy and unstable is a continuous challenge*” and “*Feeling confidence despite dizziness and unsteadiness*”, and seven categories illustrating the manifest content: Limitations in daily life; Being emotionally affected; The need for physical support; Everything takes time; I can still manage; Feelings of acceptance; and Finding individual solutions.

**Conclusions**: All participants experienced the balance limitations as a continuous challenge in their everyday life, yet they also felt confidence. They had to some degree adapted their activities and were able to manage their daily life.

## Introduction

Stroke is the leading cause of serious, long-term disability among adults (A. Pollock et al., 2014; WHO Task Force on Stroke and Other Cerebrovascular Disorders, 1989). Stroke events are either ischaemic or haemorrhagic (WHO Task Force on Stroke and Other Cerebrovascular Disorders, 1989) and a disease mainly of the ageing population. The median age in Sweden for having a first stroke is 74 years for men and 78 years for women (RIKS-STROKE TSSR, 2019). Motor impairment with muscle weakness and spasticity, impaired sensory function and cognitive deficits is common disabilities after stroke and may affect balance control (Langhorne et al., 2011; A. Pollock et al., 2014). The motor deficits contribute to weight-bearing asymmetry, which means more load on the non-paretic side, and leads to a gait with marked asymmetry (Kamphuis et al., 2013; Marigold & Eng, 2006). One consequence of the asymmetric walking pattern and the impaired balance control is a high risk for falls (Hendrickson et al., 2014; Kamphuis et al., 2013). Impaired balance affects walking and independence in activities of daily living (Langhorne et al., 2011; Van de Port et al., 2006). In daily living, several activities are performed simultaneously, which can be challenging for persons who have suffered a stroke (A. Pollock et al., 2014).

In about one-third of persons with stroke, there is a discordance between perceived and measured balance (Liphart et al., 2016). Balance control emerges from an interaction between the individual, the task and the environmental demands (Shumway-Cook & Woollacott, 2012). To control the body, a complex integration of the motor, sensory, vestibular and cognitive systems is needed (Shumway-Cook & Woollacott, 2012). Balance can be measured both subjectively and objectively; however, the results on perceived and measured balance may differ. Recent studies (Brogardh et al., 2012; Liphart et al., 2016; Robinson et al., 2011) suggest that both subjective and objective perspectives must be considered when dealing with balance problems after stroke, but most previous studies have mainly focused on the objective measures of balance (French et al., 2016; Rose et al., 2017). Qualitative studies can add to this, by capturing a person’s experiences, feelings and perceptions (Ohman, 2005). In qualitative research, an individual’s experiences can be collected as a subjective measure and this knowledge can be valuable in developing rehabilitation interventions. Such studies are, however, rare and therefore the aim of this study was to describe experiences of living with balance limitations after a first-ever stroke.

## Materials and methods

### Study design

This study has a qualitative design aiming at capturing experiences, feelings and perceptions, including data collection through individual interviews (Kvale, 1996). Data were collected in Örebro County, Sweden, from October 2016 until January 2017, and thereafter inductive qualitative content analysis was conducted (Graneheim & Lundman, 2004).

### Subjects

The participants were recruited using the Swedish Stroke Register (Riksstroke), a nationwide quality register for stroke care (Asplund et al., 2011). Inclusion criteria were: (1) living in Örebro County and having had a first-ever stroke during the last years, (2) impaired balance, but (3) maintained language function; (4) independence in toileting and dressing; and (5) ability to walk outdoors with or without a walking aid. The inclusion criteria were self-reported and gathered from the register’s 3-month follow-up questionnaire. Exclusion criteria were other self-reported disabilities that can significantly affect balance.

In order to achieve a purposive sample (Patton, 2014), aiming for heterogeneity in age and gender, individuals meeting the inclusion criteria were informed about the study by mail in blocks of 10 to 15 persons. In each block, an even distribution of men and women and of younger and older individuals was aimed for. Thereafter, they were contacted by telephone by the first author (M.A.L.), and invited to participate. A total of 45 persons received written information about the study by mail. Twenty persons initially agreed to participate, but one declined shortly afterwards for personal reasons. Therefore, 19 persons with first-ever stroke were included, nine men and ten women. The participants were living at home, and the mean time since onset of stroke was 15 months. For participants’ characteristics, see Table I.

**Table I.** Participants’ characteristics

Time since stroke, mo, mean (SD) range	15 (6.1) 4–22
Age, yrs, mean (SD) range	77 (12) 42–92
	n
Women	10
Hemiparesis, left/right	10/9
Type of stroke Intracerebral infarction Intracerebral haemorrhage	16 3
Self-reported falls in the previous 3 months No falls	7 12
Walking aid None Unilateral support Bilateral support	**Indoor Outdoor** 148 12 49
SSST, sec, median (range)	20.2 (8.8–113.3)
ABC scale, %, median (range)	67.5 (29–98)

ABC scale = Activities-specific Balance Confidence scale (Myers et al., 1996). Suggested cut-offs on the ABC scale are >80% for a high, 50–80% for a moderate and <50% for a low level of physical functioning.

SD = standard deviation; SSST = Six-Spot Step Test: the SSST comprises a timed 5 m walk involving shifting weight from one foot to the other, and single-leg standing while shoving wooden blocks (Arvidsson Lindvall et al., 2018; Nieuwenhuis et al., 2006).

The data collection took place at a venue of each participant’s choice, either in the participant’s home (n = 16) or at their primary health-care centre (n = 3).

The study was approved by the Regional Ethical Review Board, Uppsala, Sweden (2016/307 and 2016/307-1). The study was conducted according to the Helsinki Declaration regarding informed consent and confidentiality and before the data collection, the participants signed an informed consent.

### Procedure

In conjunction with the interview, background variables were collected, including demographic and medical characteristics, such as use of walking aids indoors and outdoors, and self-reported number of falls during the last 3 months. Balance was objectively assessed using the Six-Spot Step Test (SSST) (Nieuwenhuis et al., 2006). The SSST is a timed walking test originally developed for persons with multiple sclerosis (MS), and includes a dual motor task (Nieuwenhuis et al., 2006). It has been validated for persons with stroke (Arvidsson Lindvall et al., 2018). Confidence in balance was collected using the Activities-specific Balance Confidence (ABC) scale, a self-reported questionnaire (Myers et al., 1998, 1996). The first author (M.A.L.), a physiotherapist specialized in neurological physiotherapy, and at the time a PhD student, conducted the data collection. She has extensive clinical experience of working with patients with stroke and is experienced in qualitative data collection. The authors had no previous professional contact with the participants.

#### Interview

The semi-structured individual interviews (Kvale, 1996), allowed the participants to speak freely about their experiences of balance after having had a stroke. The interviews started with a broad initial question covering experiences of balance limitations in everyday life, based on the aim of the study. Thereafter, the participants were asked to describe the experience of balance, good and bad situations in daily life, emotions surrounding balance limitations and dealing with them, and, finally, whether the participants used any strategies in situations where balance was required. As the interview had a conversational tone, probing questions were asked in response to the participants’ answers and in relation to the aim of the study. All interviews were audio-recorded digitally and transcribed verbatim. The interviews lasted 20–60 minutes. After 19 interviews, no new aspects of experiences of living with balance limitations emerged and therefore no new participants were included (Sandelowski, 1995).

### Data analysis

Inductive qualitative content analysis was performed using the software program N’Vivo 11 (QSR International, Victoria, Australia). The analysis covered both the manifest and the latent content (Graneheim & Lundman, 2004), as described below:

#### Manifest analysis

1. The text was read through and listened to several times.

2. Meaning units were identified.

3. Each meaning unit was labelled with a code, still preserving the core meaning of the content.

4. The codes were sorted and abstracted into categories, illustrating the manifest content of the data.

#### Latent analysis

5. The categories were reflected on in order to identify underlying themes combining the parts into a whole. As a final step, two themes were identified.

To support trustworthiness (Graneheim & Lundman, 2004) throughout the analysis procedure, the results were repeatedly discussed and reflected upon by three of the authors (M.A.L., A.A-C. and A.F.). The three were familiar with qualitative research methodology, and had different professional backgrounds. To illustrate the content of the categories and enhance the confirmability of the findings, a few quotations are presented in the Results section (Patton, 2014).

## Results

Two themes, “*Feeling dizzy and unstable is a continuous challenge*” and “*Feeling confidence despite dizziness and unsteadiness*”, emerged from the latent analysis of the seven categories (Table II). All participants experienced the balance limitations as a continuous challenge in everyday life, yet they also felt confidence.

**Table II.** Overview of the identified themes and categories

Themes	*Feeling dizzy and unstable is a continuous challenge*	*Feeling confidence despite dizziness and unsteadiness*
**Categories**	Limitations in daily life Being emotionally affected The need for physical support Everything takes time	I can still mange Feelings of self-acceptance Finding individual solutions

### Feeling dizzy and unstable is a continuous challenge

The theme “Feeling dizzy and unstable is a continuous challenge” revolves around participants’ descriptions of their balance as a constant feeling of dizziness and unsteadiness. Balance in daily life was limited owing to muscle weakness. It was related to feelings of anxiety, insecurity and frustration. The balance impairment became more prominent when participants were walking in darkness. This theme also includes descriptions regarding the need for physical support and the experience that everything takes time.

#### Limitations in daily life

The primary cause of limitations in balance was described as muscular weakness. Cognitive limitations were another issue the participants mentioned. These were related to a need for increased alertness, as well as to fatigue and a lack of initiative, and contributed to feelings of isolation at home. Impaired balance had a negative impact on participating in social activities, such as going shopping, and also on walking longer distances: “ … *I don’t take long walks nowadays … I feel a little wobbly … because of that ‘balance business’*.” (Man, 85 years). Fear of falling and concerns over being able to stay focused in crowded areas were also described.

It happens so fast that there are suddenly too many people … Yes, I’ve realized that I’m extremely vigilant when I’m out walking, but if there are too many people … When there’s no island of calm anywhere, just movement anywhere, that’s not good. (Man, 64 years)

Feeling dizzy was another aspect of difficulty in balance, mentioned by several participants. Because of dizziness they tended to walk with their legs wide apart, and had difficulties in picking things up from the floor. They experienced dizziness when getting up at night, which was further aggravated by darkness: “ *… I can walk a little bit but sometimes you know it starts going round and round here [in the head], so I hardly dare to walk*.” (Woman, 87 years).

#### Being emotionally affected

Almost all the participants expressed feelings of anxiety and insecurity because of their impaired balance.

Say I have to walk on some uneven surface like a lawn, or say there are stones here and there sticking out of the ground, or I have to go up or down some stairs … then I can be fearful and nervous. (Woman, 76 years)

Several participants reported fear of falling. Some expressed that they felt insecure about their ability to get up again in case of a fall or were afraid of getting a fracture. There were also accounts of anxiety regarding the ability to handle different situations where there could be a risk of falls. For instance, one participant did not want to be alone with their small grandchildren and another worried about what would happen if they tripped or got bumped into when they were out in crowded places. Some imagined the embarrassment if they fell: “

… take a tumble and end up lying on the ground … and can’t get up again, that’s what I think is difficult … – both difficult and embarrassing.” (Woman, 77 years).

Some participants related that their mood was affected by their balance problems. They expressed feelings of frustration and anger: “ *… Yes, I’m a little angry that I can’t do what I used to before, because before, I could do everything by myself*.” (Woman, 90 years).

Several participants described being frustrated because of limitations caused by the balance problems, for example, being unable to go shopping or meet with friends. Tiredness and the inability to handle a lot of noise and crowded areas had a negative impact on balance. Some felt depressed because of their balance limitations: “*I used to go out walking a lot, but now suddenly it’s as if it is not important … that’s how I’m feeling … Yes, I have no interest in doing anything … feels like nothing really matters*.” (Woman, 77 years).

#### The need for physical support

About half of the participants used walking aids, such as a rolling walker or a cane, in their everyday life. Walking aids were more often used outdoors and gave the participants confidence to go out, which they might not have done without aids. A rolling walker offered support in more than one way, both when walking and as a seat when the participants were in need of a rest: “ … *it’s ideal if you want to go for a long walk. ’Cause you’ve got your ‘chair’ with you and you can just sit down and rest*.” (Man, 92 years).

The need for physical support also had to do with confidence that there was something to grab on to when feeling unstable in everyday life situations. Many participants described using the walls and furniture for support when moving around in their apartment: “ *… I grab ahold of a chair, then there’s a door post a bit further on, and then I’ve got the railing to hold on to, the rest of the way*.” (Man, 79 years). Several persons also described themselves as more unsteady when there was not enough light: in such situations, they needed more physical support: “ … *it’s pretty dark on the stairs up to the upper floor, but there is a hand rail so I hold on to that*.” (Man, 79 years).

#### Everything takes time

Some participants, most of them women, described that many of their usual activities involving balance, such as dressing, preparing meals or walking about in the apartment, were experienced in relation to the time they took up. They felt that these activities were time-consuming and that small, simple things took much longer now than before the stroke. One lady said *“Yes, I cannot even think that I’ve to hurry. I cannot hurry anymore, for example, when I know that I’m late for an appointment, or the taxi is waiting for me”* (Woman 77)

Some were frustrated, while others seemed to accept this. Most of the participants had the experience of feeling limited by the extra time they needed for these simple activities. A man expressed “*I do everything I used to do, even though it takes much more time.”* (Man 78 years). In the past, many actions had been automatic without need to reflect, but now the participants were aware of them and how long they took: “ … *get dressed, but it takes so much more time than it used to take … Things that used to be practically automatic now take time*.” (Woman, 65 years).

### Feeling confidence despite dizziness and unsteadiness

The second theme is about participants’ perceived abilities and feelings of confidence regarding their ability to still look after themselves, despite the dizziness and unsteadiness. All the participants felt limited in their ability to keep balance, yet they did, to a varying degree, actively participate in social activities. They described having accepted their new life situation and said they were confident in their ability to manage their everyday activities.

#### I can still manage

Almost all of the participants perceived that, despite experiencing dizziness and unsteadiness, their balance had improved since the stroke. They managed to take care of themselves and perform activities of daily living, such as preparing small meals, showering and light cleaning at home. However, for some, it was challenging and exhausting even to get dressed.

Some participants described themselves as stubborn and maintained that this stubbornness kept them from accepting help; and as a result, they kept doing as much as possible themselves. However, they admitted that they sometimes needed assistance. “ … *they help me, they do, but … I’m a stubborn person and I want to manage on my own for as long as I can …* ” (Man, 92 years).

To be outdoors was regarded as important, yet challenging. Several of the participants walked outdoors every day. For some, it was a daily routine they did not want to miss; others saw it as an exercise routine, which they sometimes struggled with: “ *… I have a route I usually take. And I can say that I do it 95% of every day*.” (Man, 74 years).

Some participated in social activities outside their home, and a few used public transport to go to church or participate in training sessions. Several participants went to the grocery store on their own: “ *… I go shopping, for instance; I go by myself and it’s not far … and there’s always someone to chat with*.” (Woman, 85 years). Managing to perform similar activities as before the stroke made them cope with their new life situation. Some described that activities performed outdoors in the open, such as walking in the forest or going fishing, were challenging, but they felt satisfied when they managed them.

#### Feelings of self- acceptance

Several of the participants said they felt secure and had come to accept their daily life situation after the stroke, despite their perceived balance limitations and altered physical functioning. One participant said, “*How would my life be if I abstained from everything that is who I am, so to speak”. (Man 79)*. Another participant said, “ *… You can’t say there have been problems; rather … small challenges have arisen, but nothing so significant that I was not able to deal with it*.” (Woman, 65 years). Some participants felt more comfortable at home, even if they did not feel that their balance forced them to stay at home. Rather, they were satisfied to be at home. … *” Before the stroke, I was often out and about, but now I feel satisfied just to stay at home. I’m not as interested in going out as I was before the stroke”* (Man 69). About half of the participants expressed that they were not afraid of falling and that they felt safe, despite dizziness and unsteadiness. This confidence came from the knowledge that they had support from relatives nearby, home care and the neighbourhood.

#### Finding individual solutions

Several of the participants felt that their balance limitations forced them into increased awareness, and to always be prepared for upcoming difficulties, such as a sudden spout of dizziness, an uneven surface or a dimly lit passage. However, on the whole, they felt that they had managed to find individual solutions in their everyday life:

… it’s very bright in here and we’re always turning the lights on … of course it’s pretty dark on the stairs, and I always turn the light on when I need to walk downstairs … And I’ve never done that before … plus, in the morning, especially when I’m feeling a bit stiff, I take it one step at a time using one foot at a time. (Woman, 65 years)

One participant described having a taped path in her apartment, which helped her feel safe when moving around. Another described feeling safe in the bathroom, as there was a designated corner to stand in, as well as handles on the walls and a stepwise routine that she could follow. Others described strategies of listening to the body. One participant said,

… ” Yes, I’ve become used to listening to my body, I really have … I’ve fallen so often … I have to sit down for a while … and when I want to get up again I have to stand a moment to regain my balance.” (Woman, 87 years)

Listening to the body could also involve listening to feelings of tiredness and giving oneself the opportunity to rest: “*Yes, it really tires me out … I have to lie down and sleep … exhausted in body and soul …* ” (Man, 64 years).

## Discussion

The novelty of this study is that it explores the personal experience of living with balance limitations after a first-ever stroke. This approach offers a deepened understanding of the affected individuals’ perspective, which could be of value for professionals meeting with this group, and for tailoring rehabilitation interventions. All participants experienced the balance limitations as a continuous challenge in everyday life, yet they also felt confidence.

Several participants highlighted dizziness and unsteadiness as two diverse aspects of balance. They experienced both of these as an everyday challenge. Dizziness exacerbated the participants’ feelings of unsteadiness. In previous literature, dizziness has been described as a common condition after stroke, affecting everyday life and providing a barrier to community participation (Schmid & Rittman, 2007; M. Walsh et al., 2017), but not as a central aspect of balance. Balance, or postural control, has elsewhere been described as a person’s ability to control their position in space for stability by keeping the centre of mass within the base of support. This has been said to vary depending on the task and the environment (A. S. Pollock et al., 2000). The difference between stroke-affected people’s understanding of, and problems regarding, balance and a health professional’s definition of “balance” requires attention at the clinical meeting, in order to reach a common understanding in rehabilitation practice.

Several of the participants said that dizziness affected their ability to walk longer distances or be in crowded areas. It is known that the impairments after stroke can lead to activity limitation and participation restrictions (Langhorne et al., 2011), but not that this can be due to dizziness. A general association between dizziness, walking speed and falls efficacy has been reported in older people (Lindell et al., 2019). The participants in the current study had a mean age of 77 years at their first-ever stroke, and their experiences of balance are to be interpreted in the context of their age as well as in relation to their everyday life after stroke. To counter the feelings of dizziness and to facilitate balance in everyday life, approximately half of the participants in this study used walking aids such as a rolling walker or a cane. Walking aids were more often used outdoors and gave the participants the confidence to go out. This physical support which they used when feeling unstable in everyday life situations was associated with increased confidence and contributed to their ability to partake in outdoor activities. Use of walking aids can improve the level of physical functioning and increase activity of the person with stroke, which is good, as stroke survivors often limit their physical activity post-stroke, resulting in lowered physical fitness (Billinger et al., 2012).

Fear of falling in relation to balance and walking was also experienced as a barrier to exercise and community participation, and this was also reported by Simpson et al. (2011). As mentioned previously, in clinical practice it is therefore important to consider appropriate walking aids and work with falls prevention to minimize falls and promote physical activity. However, dependence on assistive devices and increased focus on physical limitations can challenge a stroke survivor’s identity (M. Walsh et al., 2017). To elicit fears related to falls and understand the experience of falls it is suggested to talk about fear of falling (Schmid & Rittman, 2007). Walsh et al. suggest using self-management principles, peer educators, and education (M. E. Walsh et al., 2019), these approaches imply the need for care by a multidisciplinary team with different professional knowledge areas (Organised inpatient (stroke unit) care for stroke, 2013; Veerbeek et al., 2014).

Another consequence of the stroke, which affects balance, is difficulty walking in the dark, as described by several of the participants. This may have been due to cognitive deficits, as well as to affected proprioception, touch and vision, which may all affect the ability to balance (Langhorne et al., 2011; A. Pollock et al., 2014). The need for better lighting has been reported previously by older adults. They reported improved lighting as one factor in home modification to prevent fall (Tuvemo Johnson et al., 2018) so this need is not unique for stroke survivors.

The participants in this study experienced balance impairments; nevertheless, they all said that they could manage their daily life. They had found individual solutions to their balance problems, such as increased awareness and constant anticipation of upcoming difficulties. This is similar to the findings by Pallesen et al. (2019) who report that stroke survivors find that they have to deal with the impairments after the stroke, but they also learn to manage and overcome challenges by being active and participating in everyday life (Pallesen et al., 2019). Active coping strategies have a positive impact on quality of life after stroke (Lo Buono et al., 2017; Ostir et al., 2008) and subjectively experienced wellbeing is related to the ability to manage the consequences of a stroke (Lo Buono et al., 2017; Pedersen et al., 2019).

Strategies such as using the walls and furniture for support when moving around in their apartment, using the rolling walker to sit and rest on when walking longer distances, and accepting the fact that everything takes more time were described by the participants. Strategies like these can be regarded as self-management strategies, which have been defined as “an individual’s ability to manage the symptoms, treatment, physical and psychosocial consequences and lifestyle changes inherent in living with a chronic condition” (Barlow et al., 2002). According to a self-management approach, it is important in rehabilitation to focus on problems perceived by the participants themselves. In self-management programmes, the target is to enable patients to find out new ways of taking control of their daily lives by setting small goals (McKenna et al., 2015). In the current study, the stroke survivors seemed to have practised self-management by themselves when accepting their balance challenges and finding strategies to cope despite them. Coping strategies at discharge are predictive of quality of life one year later (Darlington et al., 2009). Therefore, it is important that supportive measures for coping strategies are initiated at an early stage post-stroke.

It has been recommended that rehabilitation programmes following stroke should be individualized (Langhorne et al., 2011) and the current results support this. It is important to find out the wishes and needs of each individual and consider both their pre- and their post-stroke life when planning interventions, rather than relying only on measurements of functionality and offering standardized interventions. For example, if it is important for a person to continue walking outdoors, exercises and aids that can promote such activity should be offered.

### Strengths and limitations

The aim of the present study was to describe experiences of living with balance limitations after first-ever stroke. The qualitative design was found to be adequate and an interview guide was used, which increased the dependability of the study, as this ensured that the same topics were covered in all interviews. During the data analysis, all authors were involved in the various analytical steps, which increases the confirmability and credibility of the results. The background data show that the participants represent different age, sex and functional status groups. The demographics and medical data enable the reader to judge the transferability of our findings to other contexts.

Some of the participants were 85 years and older, and this calls for some caution when interpreting the results. It is possible that some reported limitations are due to age, and not specifically to stroke. However, in the interviews, the participants compared their current everyday life to life before the stroke, which was 4–22 (mean 15) months ago. Furthermore, their self-rated safety in difficult balance situations also varied, which supports that we reached a heterogenic sample. This increases the credibility of the study.

Information about the participants’ past or ongoing rehabilitation was not registered and no specific gender differences were addressed, which can be regarded as limitations. On the other hand, almost all participants were represented in all categories, and in both themes. As all the participants in this study had had a first-time stroke, it would be of interest to study experiences of balance in persons with multiple strokes and investigate possible connections in balance rehabilitation between dizziness, fatigue and cognition.

## Conclusions

All participants experienced their balance limitations as a continuous challenge, yet they also felt confidence in managing their daily life. To plan individual rehabilitation interventions, it is essential to capture a person’s experiences and find out the wishes and needs of each individual.

## Clinical implications

- After stroke, living with balance limitations can be a continuous challenge but over time, stroke survivors might accept their new life situation.
- It is important to understand the individual wishes and needs of each stroke survivor to facilitate rehabilitation of balance and walking activity and, thus, participation in everyday life.
